# *PLOS Biology* at 20: Reflecting on the road we’ve traveled

**DOI:** 10.1371/journal.pbio.3002340

**Published:** 2023-10-04

**Authors:** Hemai Parthasarathy, Theodora Bloom, Emma Ganley

**Affiliations:** 1 X, the Moonshot Factory, Mountain View, California, United States of America; 2 BMJ, BMA House, London, United Kingdom; 3 protocols.io, Berkeley, California, United States of America

## Abstract

In this Editorial, three editors who have previously been at the helm of PLOS Biology reflect on its beginnings and evolution as a journal.

## Teaser

Why was *PLOS Biology* launched and how has it changed over the past 20 years? Three editors who have been at the helm of the journal reflect on its beginnings and evolution.

This article is part of the *PLOS Biology* 20th Anniversary Collection.

This month, as we celebrate 20 years since the launch of *PLOS Biology*, we asked 3 editors who have led the journal to discuss the idea behind its launch and how it evolved throughout their tenures. Hemai Parthasarathy, Managing Editor 2003–2007, Theodora Bloom, Chief Editor 2008–2014, and Emma Ganley, Chief Editor 2014–2019, share their experiences below.

## The first years: Hemai Parthasarathy

The Public Library of Science (then PLoS, now PLOS) was created to further the mission of open-access publishing. The argument was simple: in the digital era, when the cost of producing and sending one copy of a journal was infinitesimal, why could publication not be paid for up front, to release the fruits of publicly funded research for the benefit of the world? We created *PLOS Biology* in service of that mission and of the scientific community.

*PLOS Biology* did not have a Chief Editor at the outset. PLOS was a startup and *PLOS Biology* was our first product, so it was all hands on deck for that first issue [1]. Not because we believed anyone needed yet another journal, let alone a so-called elite scientific journal, but because the stark obstacle to persuading scientists to publish open access was the perceived lack of high-quality venues in which to do so. Scientists repeatedly said they simply could not choose any of the existing open-access options over the traditional high-profile journals because it would be career suicide. Moreover, proponents of the publishing status quo argued that paying publication fees up front was incompatible with high editorial standards. To meet these challenges, the entire PLOS team—including our founders—worked together to recruit the academic editorial board, solicit manuscript submissions, and make decisions about the process, look, and feel of the journal.

**Fig 1:**
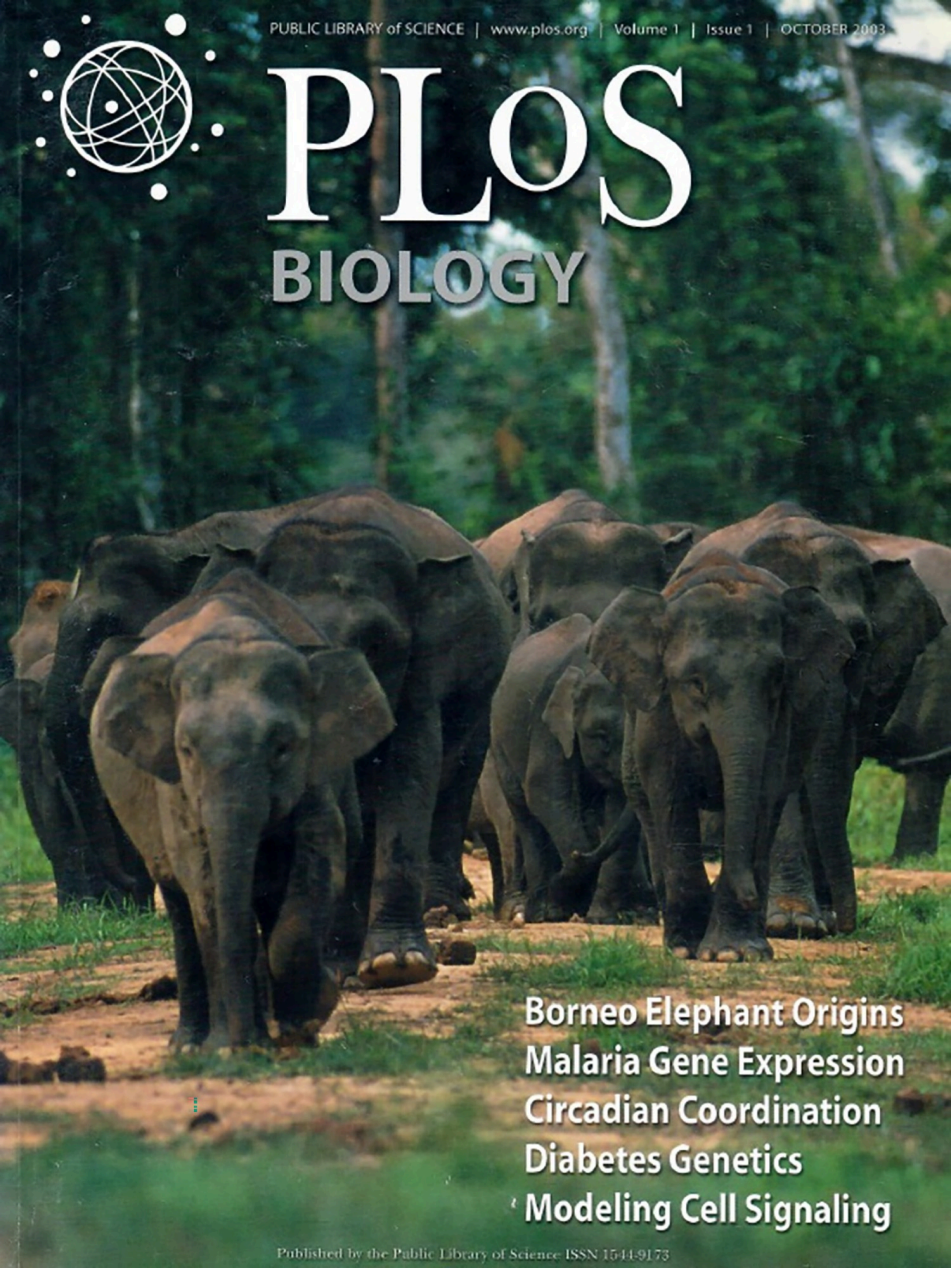
Cover of the first issue of *PLOS Biology*. Photo of native Borneo elephants kindly provided by Cede Prudente, World Wildlife Fund, Malaysia.

Twenty years is a long time. Remember, if you can, a time before Facebook, Twitter (ahem, X), and Gmail. In science publishing, AAAS had a single journal, *Science*, while Nature Publishing Group and Cell Press had short lists of titles one could easily memorize. None of them were open access. That is the world into which we launched *PLOS Biology*. We opened our digital doors with bated breath on October 13, 2003. It was as if we had spent months planning a party and now wondered if anyone would show up. We panicked when the servers crashed and then were pleased to report that it was the result of unexpectedly high traffic to our site. We printed copies of the journal for distribution at conferences, and I still aspire to travel to Borneo to see the miniature elephants that graced our first cover (Fig 1).

Once *PLOS Biology* was launched, the PLOS founding team quickly diversified our efforts and created a structure to allow us to scale and launch other journals. I took on the role of running *PLOS Biology* and hired in a team of editors dedicated to just this journal. If I am honest, I did not really care if *PLOS Biology* would exist in 20 years. In fact, I rather hoped it would not, at least in its original incarnation. I hoped that peer-to-peer scientific communication would have moved on from transposing the 300+-year-old paper journal onto a digital platform. I imagined that the line between dataset and paper would be blurred and the lines between preprint, peer-review, and validation by the community would become similarly blurred to more honestly reflect the process of scientific discovery and validation. As we were launching *PLOS Biology*, we were already working on *PLOS ONE* with those aspirations in mind. We felt that it was already impossible to keep up with the scientific literature. Open access offered a way for machines to help us, which seems prescient now that large language models and machine learning advances are galvanizing changes in the way we scientists operate, hopefully freeing us to be more creative [2].

I left scientific publishing more than 15 years ago to work with scientist entrepreneurs. Every startup founder I have supported has asked whether I could help them get access to subscription-based scientific publications, once they were no longer associated with a university. Insofar as the peer-reviewed, high-profile journal article continues to be valued, I am proud that *PLOS Biology* still produces high-quality content that is freely available to anyone with an internet connection and has helped to catalyze a world in which open-access publication is inevitable.

## After half a decade: Theodora Bloom

I joined *PLOS Biology* as Chief Editor in February 2008. The world was in the midst of a global financial crisis but PLOS was, according to its i990 forms, moving from dependence on grants to earning most of the revenue needed to cover its costs. At launch, *PLOS Biology* and *PLOS Medicine* had aimed to be prestige titles that would reassure authors who typically published in journals with one-word names and double-digit impact factors that open-access journals could deliver the same type of kudos and recognition. But that made them expensive to produce, rejecting a lot of research articles, and providing commentary and analysis in support of those articles that were published. Key to the switch in PLOS’s fortunes, therefore, was the launch of *PLOS ONE* in 2006, which became the first successful “mega journal” that aimed to publish all research that was sound, irrespective of editorial considerations of impact and importance to the field. Before joining PLOS, I had worked at BioMed Central, which had developed the idea of article processing charge (APC)-funded journals that focused solely on the soundness of the research, but had subdivided them by subject area. So, once again, PLOS showed that by changing just one aspect of publishing, it was possible to bring the community along a new path and demonstrate the feasibility of its approach.

The *PLOS Biology* team of the 2000s had at its core a fabulous group of professional editors who had worked elsewhere in life sciences publishing before PLOS, supported by superb administrative and production staff. But it was hard to cover the whole of biology with a small group of editors, even with the help of a large editorial board, so a decision was taken to appoint Jonathan Eisen as Academic Editor in Chief, to act as “a community representative for the journal both inside and outside of PLOS.” In this role Jonathan—a prolific blogger, tweeter, and author—was able to anchor the editorial team in the academic community and provide invaluable outreach to help spread the word about PLOS and open access [3].

By the 2010s, when *PLOS Biology* was celebrating its 10th anniversary, it could claim to be a mature journal and part of an ecosystem of journals published by an increasingly successful nonprofit open-access publisher. Questions were raised publicly about whether authors at *PLOS ONE* should be “subsidizing” the cost of rejections from *PLOS Biology* and *PLOS Medicine*. My recollection is that PLOS’s Board of Directors also struggled with this notion, sometimes deciding that *PLOS Biology* ought to be self-sustaining and at other times accepting that it served a different purpose in the PLOS ecosystem. This struggle was perhaps exemplified by the comments of PLOS Founder Mike Eisen at the anniversary party, that the best thing *PLOS Biology* could do in the next 10 years would be to cease to exist. That was a hard message to hear for a team who had worked to produce a distinctive journal that met its original goals and continues to play a key part in PLOS’s reputation and fortunes, as well as serving authors and readers globally.

If I were to look to the next 20 years for *PLOS Biology*, my hope might be that it would become even less focused on the USA and western Europe, even more concerned about equity and the difficulties of the APC model, and even better at including editors and authors from around the globe. It has come a long way since the days when a conference call meant clustering around a polycom phone with colleagues whose faces one never saw. I look forward to seeing how it develops next.

## Maturity and beyond: Emma Ganley

Having seen *PLOS Biology* during its infancy and teen years in 2 different stints as Associate Editor and Senior Editor working with both Hemai and Theo, respectively, it was a dream and a privilege to take the baton as Chief Editor, initially with a co-chief, Christine Ferguson, and then solo. My five-year tenure as Chief was jam-packed, working with an incredible team of professional and academic editors, colleagues across PLOS, and of course amazing researchers (thank you to all involved). There was never a dull day; it was a time of opportunity for *PLOS Biology* to delve deeper into the role of innovator.

There was an increasing focus in the research community on accountability and internal review, and more “research into research.” The meta-research field blossomed, but accessible publishing options for resulting studies were limited, so we created a much-needed “Meta-Research Article” and added appropriate experts to our Editorial Board to accommodate these research efforts [4].

Addressing researchers’ pain points was a general focus. Researchers wanted to publish initial preliminary findings, methods, and/or resources, and none of these fit under our existing Research Article rubric, and so “Short Reports” and “Methods and Resources” were added [5]. Another publishing status quo that made no sense given concerns about the reproducibility of research was the worry around “scooping.” We decided to formalize a previously undocumented policy to encourage confirmatory research. Where previously, being “scooped” was cause for despair, our complementary research policy recognized the value of replication studies and stated that they are welcome at *PLOS Biology*, acknowledging the importance of being second [6].

During my tenure, cross-PLOS projects such as a bidirectional linkage between journal submission and preprint posting at bioRxiv and the roll-out of open peer review were launched. On *PLOS Biology*, we devised an approach to publish manuscripts with contentious results that reviewers and editors could not agree on. After expert review, rejection due to contention made no sense; the authors would not get a better review process elsewhere, they would have to start from scratch, adding unnecessary delay, and the contentious issue would remain. We started to publish these studies with an Academic Editor’s note and a Primer from another expert to contextualize the contention, address limitations, and rationalize the decision. Alongside open peer review, this transparent publishing approach [7] allows the scientific community to reach an informed decision about the research.

Our “magazine” section also evolved, experimenting with new article types. Our in-house writer and editor, Liza Gross, collaborated widely and brought together some incredibly important public health and conservation-relevant collections [8,9]. Before moving on to my next gig, the groundwork for several other projects was in place, such as the launch “Preregistered Research Articles” and article types that would help to redefine selectivity and what constitutes a publishable unit. With these efforts in place, I moved on from PLOS and now focus on ensuring access to research methods as an independent research output at protocols.io.

Looking to the future, I’m interested to see whether, how, and if the publishable unit is further refined. While successfully switching from print to online, research publishing has clung to the same article format that has existed since the 1600s. Perhaps *PLOS Biology* can move towards more modular publishing, with research questions, methods, results, data, code, and interpretations available as independent but interlinked objects. I hope that *PLOS Biology* and PLOS can leverage technology to continue to innovate and explore possibilities, pushing the needle for what is considered the norm.
